# Treatment of stage IV gastric cancer with induction chemotherapy using S-1 and cisplatin followed by curative resection in selected patients

**DOI:** 10.1186/1477-7819-12-406

**Published:** 2014-12-30

**Authors:** Masaaki Saito, Hirokazu Kiyozaki, Osamu Takata, Koichi Suzuki, Toshiki Rikiyama

**Affiliations:** Department of Surgery, Saitama Medical Center, Jichi Medical University, 1-847 Amanuma-cho, Omiya-ku, Saitama, 330-8503 Japan

**Keywords:** Stage IV gastric cancer, Preoperative chemotherapy, Gastrectomy

## Abstract

**Background:**

The standard treatment for stage IV gastric cancer is chemotherapy, but outcomes remain poor. The effectiveness of induction chemotherapy followed by surgery in selected patients who had a good response to chemotherapy is unclear.

**Methods:**

A total of 59 patients with stage IV gastric cancer received induction chemotherapy with S-1 and cisplatin. In each cycle, oral S-1 (80 mg/m^2^) was administered for 3 weeks, followed by a 2-week drug holiday. Intravenous cisplatin (60 mg/m^2^) was administered on day 8 after adequate premedication and hydration. If unresectable features resolved after chemotherapy, patients underwent curative (R0) resection. The safety and outcomes of this treatment combination were evaluated, and predictive factors for survival were determined.

**Results:**

Thirteen of 59 patients (22%) were eligible for R0 resection after induction chemotherapy. Kaplan-Meier analysis showed an overall median survival time of 13 months and a 3-year survival rate of 18.2%. Among patients who underwent R0 resection, the median survival time was 53 months and the 3-year survival rate was 53.8%. Multivariate analyses showed that negative para-aortic lymph nodes and undergoing R0 resection were independent predictors of survival.

**Conclusions:**

Treatment of stage IV gastric cancer with S-1 and cisplatin induction chemotherapy followed by R0 resection is safe and may improve survival compared with chemotherapy alone. Further study of this dual-modality therapy is warranted.

## Background

According to the World Health Organization cancer statistics, gastric cancer is the second most common malignancy worldwide, and the fourth most common cause of cancer mortality [1, 2]. Surgical resection is the preferred treatment for gastric cancer, but approximately two-thirds of patients have unresectable disease at the time of diagnosis [3]. Patients with advanced disease may present with local invasion, peritoneal dissemination, hepatic metastasis, or para-aortic lymph node metastasis. Prognosis in these patients is poor, with a median survival time of 3 to 5 months without treatment [4, 5] and a reported 5-year survival rate of 9.4% [2].

The current standard treatment for stage IV gastric cancer is systemic chemotherapy [6, 7]. The prognosis has gradually improved because of advances in chemotherapy regimens, but is not yet satisfactory, and permanent cure is rarely achieved. In Japan, combination induction chemotherapy using S-1 and cisplatin is the current standard treatment for unresectable gastric cancer, based on the results of the SPIRITS (S-1 and cisplatin versus S-1 alone for first-line treatment of advanced gastric cancer) trial [8].

Recently, various multimodality therapies including chemotherapy, radiotherapy, and surgery have been investigated in an effort to improve outcomes. For resectable tumors, preoperative chemotherapy reduces tumor size, treats micrometastases, and increases the rate of curative (R0) resection. Some authors have reported treatment of stage IV gastric cancer patients with chemotherapy followed by surgery [9–15], but the efficacy of combining current chemotherapy regimens with surgery in selected patients remains unclear.

This study reviewed patients with stage IV gastric cancer who were treated with induction chemotherapy using S-1/cisplatin followed by R0 resection in selected patients who responded well to chemotherapy, to evaluate the safety and outcomes of this treatment combination, and identify predictive factors for survival.

## Methods

### Inclusion and exclusion criteria

Patients with unresectable stage IV gastric cancer who were treated at the Saitama Medical Center, Jichi Medical University, Japan from January 2008 to March 2010 were retrospectively reviewed. Inclusion criteria for the study were: (1) gastric cancer diagnosed by upper gastrointestinal endoscopy and histologically confirmed as adenocarcinoma; (2) unresectable features detected on thoracic, abdominal, or pelvic multidetector computed tomography (CT) scan; (3) ability to maintain an oral liquid or solid intake without vomiting; (4) Eastern Cooperative Oncology Group Performance Status (ECOG PS) 0 or 1; and (5) white blood cell count ≥ 3,000/ml, platelet count ≥100,000/ml, serum creatinine ≤ 1.2 mg/dl, aspartate aminotransferase (AST) < 70 IU/l, and alanine aminotransferase (ALT) < 70 IU/l. Unresectable features were defined as: invasion of the primary lesion into adjacent organs (T4), ascites or peritoneal nodules (P1), para-aortic lymph node metastasis (N3), bulky lymph node metastasis or invasion into the area around the celiac trunk (bulky N2), or hepatic metastasis (H1). Exploratory laparoscopy was recommended for diagnosis of peritoneal dissemination, but was not mandatory. Peritoneal dissemination was diagnosed by imaging by a specialist in radiological interpretation. Exclusion criteria were: (1) prior history of gastric cancer treatment; and (2) known distant metastasis to sites other than the lymph nodes, liver or peritoneum. Patients were not routinely investigated for distant metastasis, but those with symptoms suggestive of metastasis underwent appropriate investigations. The gross and histological tumor types, depth of tumor invasion, and lymph node metastasis (TNM) stage were described according to the Japanese Classification of Gastric Carcinoma (JCGC; 13th edition) of the Japanese Gastric Cancer Association [16]. This study was approved by the Research Ethics Committee at Jichi Medical University.

### Induction chemotherapy and evaluation of response

All patients received induction chemotherapy using S-1 and cisplatin according to the protocol described in the SPIRITS trial [8]. In each cycle, oral S-1 (80 mg/m^2^) was administered for 3 weeks followed by a 2-week drug holiday. Intravenous cisplatin (60 mg/m^2^) was administered on day 8 after adequate premedication and hydration.

Complete blood cell count, and serum creatinine, total bilirubin, AST and ALT levels were measured before each cycle and regularly during each cycle. Chemotherapy toxicity was evaluated according to the National Cancer Institute Common Toxicity Criteria for Adverse Events (NCI CTCAE) version 3.0. If grade 4 leukopenia or grade 3 or 4 thrombocytopenia were observed, the S-1 and cisplatin doses were reduced by 1 dose level. If the serum creatinine level exceeded 1.5 mg/dl, cisplatin was discontinued and S-1 monotherapy was continued.

Response was assessed after every two cycles of chemotherapy. Measurable tumors were evaluated using the Response Evaluation Criteria in Solid Tumors (RECIST) [17]. The best overall response was evaluated and the response was not confirmed for 4 weeks. The primary gastric lesion was assessed using endoscopy, gastroduodenal barium contrast study, or CT scan according to the JCGC criteria.

### Surgical intervention and follow-up chemotherapy

If the unresectable features were resolved at the time of reassessment, surgery was performed 4 to 6 weeks after completion of S-1 administration. Informed consent was obtained from all patients. If surgical exploration in these patients did not reveal unresectable features, R0 resection was performed. Patients underwent total or distal gastrectomy according to the location of the gastric tumor with D2 lymph node dissection, plus extended para-aortic lymph node dissection (PAND) if they were para-aortic lymph node-positive (cN3) at presentation. If surgical exploration revealed unresectable features, exploratory laparotomy or bypass surgery was performed. Postoperative follow-up included blood testing every 3 months and abdominal ultrasonography every 6 months. Patients also underwent yearly abdominal CT scans and upper gastrointestinal endoscopy.

Response to induction chemotherapy, surgical procedure, extent of lymph node dissection, intraoperative blood loss, postoperative complications, pathological examination, and final disease stage were recorded. The therapeutic response was graded according to histological features. Survival was defined as the time from the start of induction chemotherapy until death or the last follow-up. Survival data were updated to December 2013.

Patients who underwent R0 resection received S-1 alone as postoperative adjuvant chemotherapy for 1 year. The remaining patients continued to receive S-1 and cisplatin therapy until disease progression was evident. When postoperative recurrence was found or the tumor was refractory to S-1 and cisplatin, irinotecan monotherapy (100 mg/m^2^ intravenously on days 1, 8 and 15) was administered as second-line treatment and paclitaxel monotherapy (80 mg/m^2^ intravenously on days 1, 8 and 15) was administered as third-line treatment, and continued until an adverse effect occurred or further disease progression was confirmed. Twenty-nine patients received second-line treatment and 12 received third-line treatment.

### Statistical analysis

Overall survival rates for the whole group and for the R0 resection group were analyzed using the Kaplan-Meier method. Prognostic factors for survival were analyzed with univariate and multivariate analyses using the Cox proportional hazards model. All reported *P-*values were 2-sided, and *P* < 0.05 was considered to be statistically significant. Statistical analyses were performed using SPSS version 22.0 (SPSS, Chicago, IL, USA).

## Results

### Patient characteristics

Of the 88 patients with stage IV gastric cancer who were treated at our institute during the study period, 59 (45 male and 14 female) met the inclusion criteria. The median age of patients was 65 years (40 to 74 years). ECOG PS was 0 in 46 patients and 1 in 13 patients. Thirty patients had differentiated adenocarcinoma and 29 had undifferentiated adenocarcinoma. Preoperative laparoscopy was performed in 10 patients, but the results did not influence subsequent management. The frequencies of features that deemed the tumor to be unresectable are shown in Table 1.

**Table 1:** **Factors making tumors unresectable at baseline**

Factor	***N***
Direct invasion into adjacent organs	9
Para-aortic lymph node metastasis	21
Bulky N2 lymph nodes	7
Liver metastasis	7
Peritoneal metastasis	26

### Clinical response to induction chemotherapy

According to the RECIST criteria, 35 of the 59 patients had metastatic lesions detected on abdominal CT scan before induction chemotherapy (lymph node metastasis in 28 patients and liver metastasis in 7). Of these 35 patients, 13 (37.1%) responded to chemotherapy (9 patients had a partial response and 4 had a complete response). Overall, patients received 1 to 8 cycles (median 4 cycles) of induction chemotherapy with S-1 and cisplatin. Patients who underwent surgery received 2 to 10 cycles (median 2 cycles) of induction chemotherapy (Table 2).

**Table 2:** **Details of the 13 patients who underwent R0 resection**

Case	Unresectable factor	Induction chemotherapy (course)	Final stage	Postoperative chemotherapy	Survival	Relapsed site
1	N3	3	IV	Paclitaxel	Dead	LN
2	N3	2	IIIB	S-1	Alive	-
3	N3	2	IV	UFT	Dead	LN
4	T4	2	IIIA	S-1	Alive	-
5	T4	2	IV	-	Dead	P
6	BulkyN2	2	IIIB	S-1	Alive	-
7	BulkyN2	2	IIIB	-	Alive	-
8	N3	2	IV	UFT	Dead	LN
9	BulkyN2	2	IIIB	S-1	Alive	-
10	P1	3	IV	-	Dead	P
11	P1	6	IIIA	S-1	Alive	-
12	BulkyN2	2	IIIA	-	Alive	-
13	P1	10	IV	-	Dead	P

H1, hepatic metastasis; LN, lymph nodes; bulky N2, bulky lymph node metastasis or invasion into the area around the celiac trunk; N3, para-aortic lymph node metastasis; P, peritoneum; P1, ascites or peritoneal nodules; T4, invasion of the primary lesion into adjacent organs, UFT, tegafur- uracil.

According to the JCGC criteria, 30 of the 59 patients (50.8%) responded to chemotherapy. The primary lesion showed a partial response in 28 patients and a complete response in 2. Table 3 shows the adverse effects of induction chemotherapy treatment. Nineteen patients (32.2%) had grade 3 adverse effects, and no patients had grade 4 effects. There were no treatment-related deaths.

**Table 3:** **Adverse effects of induction chemotherapy**

	Toxicity grade (NCI-CTC)				Total (%)	Grade 3/4 (%)
	1	2	3	4		
Leukopenia	8	17	4	0	49.2	6.8
Neutropenia	2	15	0	0	28.8	0
Thrombocytopenia	6	2	0	0	13.6	0
Anemia	7	5	2	0	23.7	3.4
Anorexia	21	11	0	0	54.2	0
Nausea	15	6	8	0	49.2	13.6
Vomiting	6	4	3	0	22.0	5.1
Diarrhea	7	1	2	0	16.9	3.4

*Abbreviation*: National Cancer Institute Common Toxicity Criteria for Adverse Events.

### Surgical outcomes

Sixteen patients were deemed eligible for surgery because of resolution of the factors that had made their cancer unresectable. Of these sixteen patients, four presented with cN3, four with bulky N2, six with cP1, and two with cT4 cancer. Of the six patients with cP1, three were found to have residual peritoneal seeding at the time of surgery, which made them ineligible for R0 resection. Two of these three underwent bypass surgery only and one underwent exploratory laparotomy only. The remaining 13 patients underwent R0 resection, resulting in an R0 resection rate of 22% (13/59). The details of surgery are shown in Table 4. We found no correlation between histological response and clinical outcome.

**Table 4:** **Details of the 16 patients who underwent surgery**

Peritoneal cytology	***N***
Negative	13
Positive	3
Type of resection	
Total gastrectomy	5
Distal gastrectomy	8
Bypass	2
Exploratory laparotomy	1
Dissection of lymph nodes	
D2	5
D2 + para-aortic	8
Operative time (minutes), range	235 (40 to 320)
Blood loss (ml) , range	210 (85 to 550)
Blood transfusion	1

Of the 13 patients who underwent R0 resection, 2 (15%) developed postoperative complications. According to the NCI CTCAE, grade 2 wound infection and grade 3 intra-abdominal abscess due to a pancreatic fistula occurred in 1 patient each. There was no postoperative in-hospital mortality.

### Patient survival

**Figure 1:**
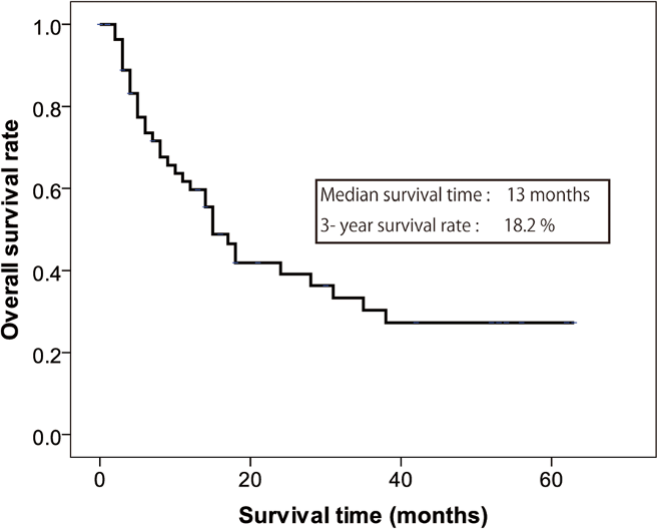
**Kaplan-Meier curve of overall survival (** ***n*** **= 59).**

At the time of analysis in December 2013, 34 of the 59 patients had died, and the median follow-up period of the remaining 25 patients was 26 months (range 1 to 63 months). Kaplan-Meier analysis showed an overall median survival time of 13 months and a 3-year survival rate of 18.2% (Figure 1). Multivariate analyses identified negative para-aortic lymph nodes (hazard ratio (HR) 0.086, 95% confidence interval (CI) 0.020 to 0.383, *P* = 0.002) and undergoing R0 resection (HR 0.285, 95% CI 0.131 to 0.623, *P* < 0.001) as independent prognostic factors for survival (Table 5). Age, sex, T stage, bulky N2, peritoneal metastasis, distant metastasis, response according to the JCGC criteria, and second-line chemotherapy were not significantly associated with survival.

**Table 5:** **Results of multivariate analyses of associations between patient characteristics and survival (Cox proportional hazards model)**

Variable		***P***
R0 surgery	(+/–)	< 0.001
Para-aortic lymph node metastasis	(negative/positive)	0.002
Age (years)	(≤59/≥ 60)	0.790
Sex	(male/female)	0.115
T stage	(T2, T3/T4)	0.936
Bulky N2	(-/+)	0.855
Peritoneal metastasis	(P0/P1)	0.358
Liver metastasis	(H0/H1)	0.125
Distant metastasis	(M0/M1)	0.913
Response (JCGC criteria)	(CR, PR/SD, PD)	0.182
Second-line chemotherapy	(+/–)	0.546

*Abbreviations*: *CR* complete response, *PD* progressive disease, *JCGC* Japanese Classification of Gastric Carcinoma, *SD* stable disease, *PR* partial response.

**Figure 2:**
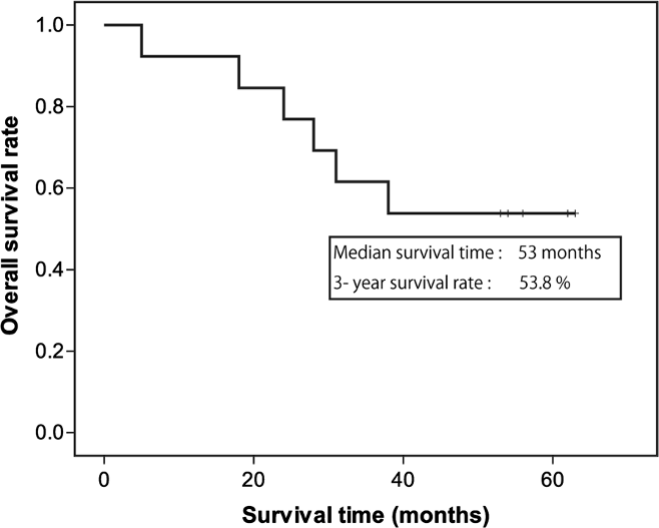
**Kaplan-Meier curve of survival in patients who underwent R0 resection (** ***n*** **= 13).**

Among the patients who underwent R0 resection, six developed recurrence (three with para-aortic lymph node, and three with peritoneal dissemination). All patients with recurrence received chemotherapy but they all died: one each with lung metastasis at 15 months, brain metastasis at 12 months, liver metastasis at 31 months, lymph node recurrence at 36 months, peritoneal dissemination at 24 months, and peritoneal dissemination at 28 months) (Table 2). Kaplan-Meier analysis of patients who underwent R0 resection showed a mean survival time of 53 months and a 3-year survival rate of 53.8% (Figure 2).

## Discussion

In Japan, S-1 and cisplatin combination chemotherapy is the standard treatment for stage IV gastric cancer, based on the results of the SPIRITS trial [8]. Although the selection of chemotherapy regimen in P1 patients requires particular care because of potential gastrointestinal symptoms and toxicity, S-1 and cisplatin combination therapy was considered to be appropriate for the patients in this study because such patients were also included in the SPIRITS trial. As shown in Table 3, serious adverse events associated with induction chemotherapy occurred at rates similar to those in the SPIRITS trial. The SPIRITS trial showed a median overall survival time of 13.0 months, which is similar to the median overall survival time of 15.0 months in the present study.

In patients with resectable gastric cancer, preoperative chemotherapy has the following advantages: (1) possible prolonged survival and recurrence-free survival due to eradication of micrometastases; (2) antitumor effects on the primary lesion and lymph node metastases; (3) down-staging, allowing less-invasive surgery; (4) better compliance than with postoperative chemotherapy; (5) superior drug delivery and penetration in the presence of tumor-feeding blood vessels and lymphatic channels; (6) clarification of the sensitivity of the tumor to chemotherapeutic agents; and (7) confirmation of the presence or absence of new lesions [18]. These advantages raise the possibility that some patients who respond well to chemotherapy may benefit from subsequent R0 resection. It is clear that a combination of surgery and chemotherapy is advantageous in resectable gastric cancer, but the outcome of this dual-modality treatment in stage IV gastric cancer treated with S-1 and cisplatin chemotherapy is still unclear. There is concern that chemotherapy may cause an increase in the incidence of postoperative complications, that post-chemotherapeutic changes may interfere with tissue dissection, and that accurate staging before chemotherapy is difficult.

In the present study, 22% of patients were deemed eligible for R0 resection after chemotherapy. The only severe postoperative complication was an intra-abdominal abscess due to a pancreatic fistula (grade 3) in one patient. The operative time and blood loss were comparable to those of conventional R0 resection, suggesting that gastrectomy can safely be performed after induction chemotherapy. Previously reported cases of patients who underwent gastrectomy following S-1 and cisplatin induction chemotherapy had similar results [9–13]. In the present study, patients who underwent R0 resection had a 3-year survival rate of 53.8%, which is similar to that reported in a previous study of stage IV gastric cancer patients who received chemotherapy followed by surgery [13].

The REGATTA study is an ongoing phase III controlled trial comparing gastrectomy + postoperative chemotherapy and chemotherapy alone. Interim analysis showed that overall survival of the gastrectomy + postoperative chemotherapy group was lower than that of the chemotherapy alone group (HR: 1.08, 95% CI: 0.74 to 1.58). Debulking surgery for gastric cancer was unsuccessful, except when it aimed for R0 resection.

The JCOG9501 trial showed that prophylactic D2 node dissection + PAND in patients with negative para-aortic lymph nodes did not improve 3- and 5-year survival rates compared with D2 node dissection alone, leading to the conclusion that prophylactic PAND should not be performed for curable advanced gastric cancer [19]. However, the JCOG9501 trial did not include data on patients who were positive for para-aortic lymph nodes who had received induction chemotherapy. Further studies are therefore needed to clarify the significance of PAND following induction chemotherapy.

In the present study, multivariate analyses identified negative para-aortic lymph nodes and undergoing R0 resection as independent prognostic factors for survival. However, para-aortic lymph node status cannot be definitively determined preoperatively, and therefore cannot be used to determine which patients are likely to benefit from R0 resection. Prolonged survival times were expected in the group undergoing R0 resection, because this group had the best response to chemotherapy. Overall survival in the present study was similar to that reported in the SPIRITS trial, thus, it is not yet clear if adding surgery to the treatment protocol provides a survival benefit. Further studies with larger patient numbers and longer follow-up periods are required to evaluate this.

A number of limitations of this study should be discussed. This was a retrospective review of patients who received treatment, and there was no control group for comparison. Patient numbers were small, and long-term follow-up was not available. In this study, 26 patients had peritoneal metastasis. However, laparoscopy was performed only in 10 patients for various reasons. Peritoneal dissemination was diagnosed in the other 16 patients by imaging by a specialist in radiological interpretation. However, without laparoscopy, it is not easy to determine whether peritoneal metastases are present clinically. It seems that staging laparoscopy is necessary for preoperative precise diagnosis of peritoneal dissemination. There was considerable heterogeneity among the patients in our study. However, our results suggest that surgery is safe, and that the possibility of prolonged survival after surgery should be further investigated. Further studies with longer term follow-up are needed to assess the safety and effectiveness of this combination therapy.

## Conclusions

Treatment of stage IV gastric cancer with S-1 and cisplatin induction chemotherapy followed by R0 resection is safe and may improve survival compared with treatment with chemotherapy alone. Further study of this dual-modality therapy is warranted.

## Abbreviations

ALT: alanine aminotransferase
AST: aspartate aminotransferase
CR: complete response
CT: computed tomography
ECOG PS: Eastern Cooperative Oncology Group Performance Status
HR: hazard ratio
JCGC: Japanese Classification of Gastric Carcinoma
JCOG: Japanese Clinical Oncology Group
NCI CTCAE: National Cancer Institute Common Terminology Criteria for Adverse Events
PAND: para-aortic lymph node dissection
PD: progressive disease
PR: partial response
R0: curative resection
RECIST: Response Evaluation Criteria in Solid Tumors
S-1: tegafur-gimeracil-oteracil potassium, UFT, tegafur- uracil.
